# A BERT-GNN Approach for Metastatic Breast Cancer Prediction Using Histopathology Reports

**DOI:** 10.3390/diagnostics14131365

**Published:** 2024-06-27

**Authors:** Abdullah Basaad, Shadi Basurra, Edlira Vakaj, Ahmed Karam Eldaly, Mohammed M. Abdelsamea

**Affiliations:** 1 School of Computing and Digital Technology, Birmingham City University, Birmingham B4 7XG, UK; abdullah.basaad@mail.bcu.ac.uk (A.B.); shadi.basurra@bcu.ac.uk (S.B.); edlira.vakaj@bcu.ac.uk (E.V.); 2 Department of Computer Science, University of Exeter, North Park Road, Exeter EX4 4QF, UK; a.karam@ucl.ac.uk

**Keywords:** LLM, GNN, XAI, node classification, MBC, extra trees classifier, univariate selection, random forest classifier, BERT

## Abstract

Metastatic breast cancer (MBC) continues to be a leading cause of cancer-related deaths among women. This work introduces an innovative non-invasive breast cancer classification model designed to improve the identification of cancer metastases. While this study marks the initial exploration into predicting MBC, additional investigations are essential to validate the occurrence of MBC. Our approach combines the strengths of large language models (LLMs), specifically the bidirectional encoder representations from transformers (BERT) model, with the powerful capabilities of graph neural networks (GNNs) to predict MBC patients based on their histopathology reports. This paper introduces a BERT-GNN approach for metastatic breast cancer prediction (BG-MBC) that integrates graph information derived from the BERT model. In this model, nodes are constructed from patient medical records, while BERT embeddings are employed to vectorise representations of the words in histopathology reports, thereby capturing semantic information crucial for classification by employing three distinct approaches (namely univariate selection, extra trees classifier for feature importance, and Shapley values to identify the features that have the most significant impact). Identifying the most crucial 30 features out of 676 generated as embeddings during model training, our model further enhances its predictive capabilities. The BG-MBC model achieves outstanding accuracy, with a detection rate of 0.98 and an area under curve (AUC) of 0.98, in identifying MBC patients. This remarkable performance is credited to the model’s utilisation of attention scores generated by the LLM from histopathology reports, effectively capturing pertinent features for classification.

## 1. Introduction

Metastatic breast cancer (MBC) represents a crucial challenge in the realm of oncology and is the main cause of death for patients with breast cancer. It needs a significant contribution to the burden of cancer-related mortality among women worldwide [1]. Despite advances in detection and treatment modalities, the metastatic spread of breast cancer remains a critical clinical challenge, necessitating innovative approaches to improve early identification and intervention [2]. Metastasis, the process by which cancer cells disseminate from the primary tumour site to distant organs or tissues, represents a crucial stage in cancer progression. Statistics underscore the gravity of metastatic breast cancer, with approximately 30% of early-stage breast cancer cases eventually progressing to metastatic disease [3]. Moreover, MBC accounts for the majority of breast cancer-related deaths, highlighting the urgent need for improved diagnostic and therapeutic strategies.

Cancer is a multifaceted disease that presents significant challenges in terms of diagnosis and management. Proper diagnosis is essential for devising effective treatment strategies and improving patient outcomes. Historically, histopathological examination has played a central role in cancer diagnosis, providing valuable information on cellular and tissue-level changes associated with malignancies [4,5]. Despite the onset of molecular and genomic techniques, histopathology remains indispensable in cancer diagnosis and management, providing complementary information that contributes to a comprehensive understanding of the disease [6]. Integrating histopathological data with molecular profiles and imaging modalities enables a holistic approach to cancer prognosis, facilitating personalised treatment strategies tailored to individual patient characteristics [7]. Histopathology involves the microscopic examination of tissue specimens to assess their morphological characteristics, including cell morphology, tissue architecture, and the presence of pathological abnormalities such as tumour formation [8]. This analysis enables pathologists to identify the type of cancer, assess its aggressiveness or grade, determine its stage, and provide crucial prognostic information for treatment planning [9]. Over the years, histopathological techniques have evolved significantly, with advancements in staining methods, imaging technologies, and molecular diagnostics enhancing the accuracy and precision of cancer diagnosis [10].

Histopathological reports serve as indispensable tools in the prognosis and management of cancer, including metastatic breast cancer. These reports offer detailed insights into the histological characteristics of tumour specimens, providing crucial information regarding tumour type, grade, hormone receptor status, and the presence of metastasis. The comprehensive analysis of histopathological findings plays a pivotal role in guiding treatment decisions and prognostic assessments for patients with metastatic breast cancer [11].

Leveraging the power of large language models (LLMs), such as bidirectional encoder representations from transformers (BERT), presents a novel avenue for extracting meaningful insights from histopathology reports. Using sophisticated natural language processing techniques, LLMs can describe complex textual data, extracting relevant features and contextual information crucial to cancer prognoses [12]. Integrating LLMs into the prognostic pipeline holds promise for enhancing the accuracy and efficiency of metastatic breast cancer detection.

**Figure 1:**
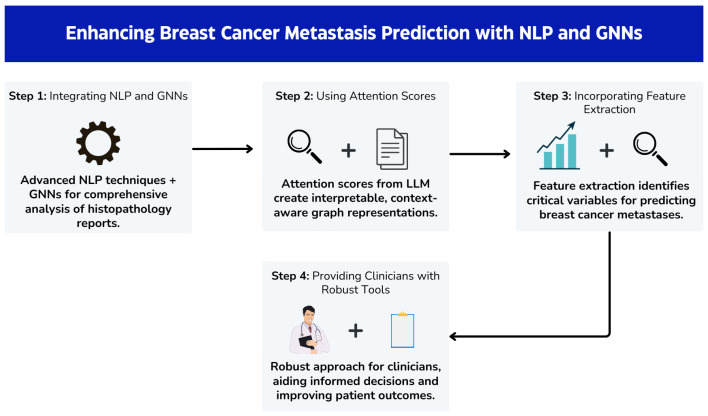
Enhancing breast cancer metastasis prediction with NLP and GNNs.

This work combines LLMs with graph neural networks (GNNs) and offers a robust framework for cancer prediction tasks, using embeddings that capture the semantic meaning of words and phrases in histopathological reports. This means that words with similar meanings or contexts are likely to have similar embeddings, even if they appear in different parts of the report, and GNNs can uncover hidden patterns and dependencies crucial for accurate disease prediction [12]. The application of GNNs in conjunction with LLMs represents a complementary approach to metastatic breast cancer detection, harnessing textual and structural information for improved prognostic accuracy [13]. Although this proposed system offers an initial step towards improving metastatic breast cancer prediction, further validation and refinement are imperative to confirm its efficacy and reliability. Subsequent investigations, including clinical trials and real-world validation studies, will be essential to assess the system’s performance in diverse patient populations and clinical settings, ultimately paving the way for its integration into routine clinical practice. Thus, the main contributions of this work can be summarised as shown in Figure 1.

1. Integrating advanced natural language processing (NLP) techniques with graph neural networks (GNNs) and proposing a novel approach for comprehensive analysis of histopathology reports.
2. Using attention scores from LLMs to construct interpretable and context-aware graph representations.
3. Incorporating feature extraction methods to identify critical variables for predicting breast cancer metastases, which enhances the model’s predictive accuracy and interoperability.
4. Providing clinicians with a robust approach that can provide valuable insights into the underlying data, thus facilitating more informed decision-making and improving patient outcomes.

The remainder of this article is organised as follows. Section 2 provides an overview of relevant studies on metastatic diseases. Section 3 details the proposed methodology of this study, including the utilisation of bidirectional encoder representations from transformers (BERT) and their application in analysing and gaining insights from histopathology reports. This section also discusses the use of attention mechanisms to extract information related to specific entities within the dataset. Additionally, it covers the refinement of attention to facilitate graph construction and feature extraction processes. Section 3.7 presents the architecture of the BG-MBC model, including the neural network structures and various layers employed in the graph neural network. This section also discusses the model training process and its capability for interpretable predictions. Section 3.8 is dedicated to the integration of the BG-MBC model with a large language model (LLM) approach. Following that, Section 3.9, Section 3.10 and Section 3.11 discuss graph building, baseline models, and the architecture of the BG-MBC model, respectively. The results section, Section 4, includes data pre-processing, comparisons with existing approaches, and model evaluation. Finally, Section 5 and Section 6 present the discussion and conclusions, respectively.

## 2. Related Work

In recent years, the application of machine learning (ML) models has gained considerable traction in the field of oncology, particularly in the context of predicting metastases and improving patient outcomes. Several studies have investigated the use of ML techniques to tackle the challenges associated with metastatic disease. One notable study by Esteva et al. [14] employed deep learning algorithms to analyse histopathology images and predict the presence of metastatic breast cancer in lymph nodes with high accuracy. The study demonstrated the potential of convolutional neural networks (CNNs) in detecting metastatic disease from digitised tissue slides, offering a promising approach for automated histopathological analysis. Liu et al. [15] proposed an automated framework using a convolutional neural network (CNN) to detect and localise tumours in gigapixel microscopy images, achieving state-of-the-art results on the Camelyon16 dataset. The method detects 92.4% of tumours at eight false positives per image, outperforming the previous best (82.7%) and human pathologists (73.2%). They achieved over 97% AUC scores and identified mislabelling in the dataset, potentially reducing false negative rates significantly. In addition to histopathological analyses, researchers have explored the utility of clinical data and molecular markers to predict metastatic outcomes. For example, Li et al. [16] developed a predictive model for distant metastasis in breast cancer patients using a combination of clinical features and gene expression profiles. The study achieved accurate prognostic predictions by integrating machine learning algorithms with gene expression data, allowing the early identification of patients at high risk of metastasis. Another study [17] evaluates various predictive models for determining the time to tumour recurrence in breast cancer patients, achieving an accuracy of up to one year. Analysing 198 patients, it was found that 40% were predicted to experience tumour recurrence within the first year of diagnosis. The study employed several classification models, including spectral clustering, DBSCAN, and k-means, alongside prediction models such as support vector machines (SVMs), decision trees, and random forests. The results highlighted the efficacy of these machine learning techniques, with SVMs achieving the highest accuracy at 78.7% in predicting the time to tumour recurrence or patient recovery. In [18], the authors proposed a multimodal deep learning method that integrates whole-slide H&E images (WSIs) and clinical data to assess relapse and metastasis risk in HER2-positive breast cancer patients. The images, resized to 512 × 512 pixels, were analysed using a deep convolutional neural network (CNN) to extract features, which were combined with clinical data. The model achieved an AUC of 0.76 in two-fold cross-validation and 0.72 on independent testing with TCGA data, demonstrating its potential for prognosis prediction despite demographic and experimental differences.

Previous studies have shown significant limitations and deficiencies, highlighting the need for a more robust approach. For instance, one study analysed 198 breast cancer patients [17] and used various machine learning techniques, such as spectral clustering, DBSCAN, k-means, SVMs, decision trees, and random forests, achieving a maximum accuracy of 78.7% with SVMs for predicting tumour recurrence within one year. However, this study was limited by a small sample size and moderate prediction accuracy, indicating the necessity for more reliable and scalable methods.

Another study used a multimodal deep learning approach [18] that integrated whole-slide H&E images (WSIs) and clinical data to predict relapse and metastasis in patients with HER2-positive breast cancer. Although it achieved promising AUC scores of 0.76 in cross-validation and 0.72 on independent TCGA data, there were still challenges in its generalisability across different patient demographics and experimental setups. These studies did not fully exploit the potential of integrating advanced language models and graph neural networks to enhance predictive power and interoperability.

Here, our proposed BG-MBC model addresses these limitations by combining large language models (LLMs), specifically the BERT model, with graph neural networks (GNNs) to analyse histopathology reports for metastatic breast cancer (MBC) prediction. By leveraging BERT embeddings to capture semantic information and constructing nodes from patient medical records, our model integrates graph information derived from BERT. It employs univariate selection, extra trees classifier, and Shapley values to identify the most crucial features of 676 generated embeddings, enhancing predictive accuracy.

## 3. Materials and Methods

**Figure 2:**
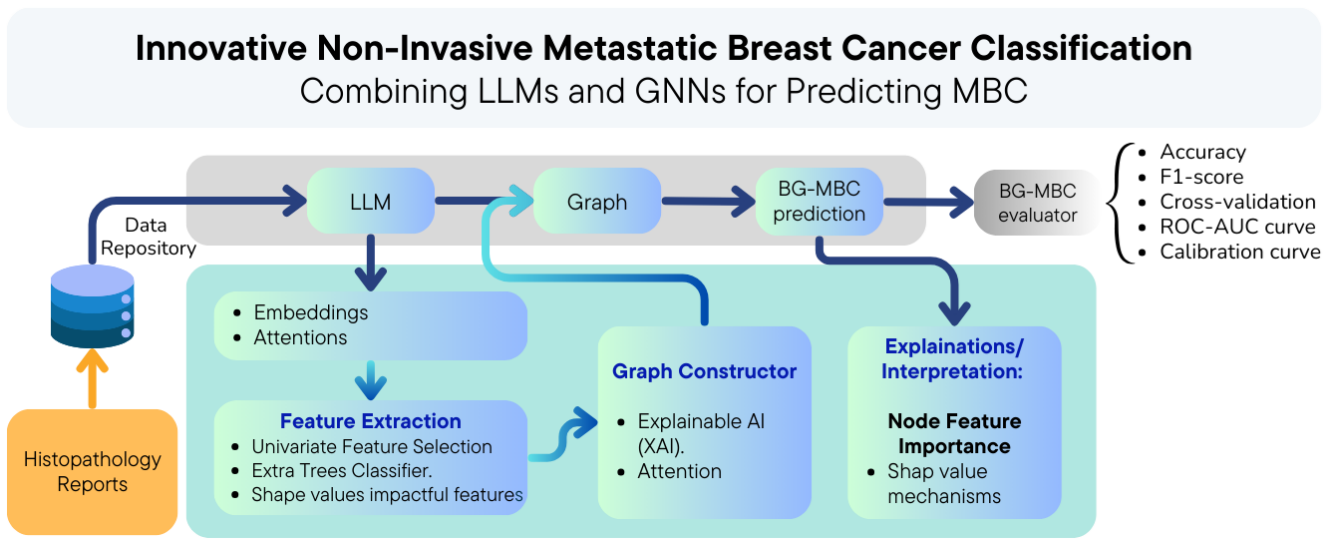
The BG-MBC model employs natural language processing (NLP) to extract crucial features from histopathology reports. Attention mechanisms construct a graph from these embeddings, which is then processed by a graph neural network (GNN). Shapley values are used to interpret and explain its predictions.

Our proposed approach leverages bidirectional encoder representations from transformers (BERT) to extract attentions and embeddings from histopathology reports, as shown in Figure 2. The attentions are utilised to define graph edges, capturing the relationships between different entities acted by medical numbers. Meanwhile, the embeddings extracted from the reports serve as node features within the graph. Combining these attentions and embeddings, a graph was constructed to represent the histopathology data. Subsequently, this graph representation was passed into the BERT graph metastatic breast cancer (BG-MBC) model. This model is designed to handle graph-structured data and is capable of effectively incorporating the complex relationships captured by the graph edges and node features. By utilising the attentions and embeddings output by BERT as inputs to the BG-MBC model, the aim is to enhance the performance of breast cancer metastasis prediction, leveraging the rich contextual information provided by the histopathology reports.

### 3.1. Dataset

Data were acquired from BIACH and RI in a semi-structured Excel file comprising two main columns: patient identifiers (medical record number—MR No) and histopathology reports (Hist_report). The dataset consists of 25,652 entries in .csv format (raw text). The Hist_report column includes a variety of pathological observations, such as clinical details, specimen descriptions, microscopic observations, impressions, and gross findings [19]. The histopathology diagnoses pertain to metastasis, biopsy, and lymph node reports. This paper focuses on detecting metastasis in patients among the different types of diagnoses.

### 3.2. Data Pre-Processing

Various data repositories have been used, containing medical records, histopathology reports, and corresponding diagnostic results. These datasets were merged into a single file to facilitate streamlined analysis. Due to the unstructured nature of the original datasets, which span multiple CSV files, it became imperative to consolidate the data into a single file for streamlined analysis. Leveraging Google Collab during the pre-processing stage, the data are formatted into a tabular structure, facilitating the merging process to consolidate all necessary information into one comprehensive dataset. Following this, extensive cleaning procedures were executed, which involved feature selection and normalisation. Feature selection aids in identifying the most informative variables for metastasis prediction, thereby reducing dimensionality and enhancing model performance. On the other hand, normalisation is crucial in ensuring that features are uniformly scaled, mitigating bias issues from differing magnitudes across variables.

**Figure 3:**
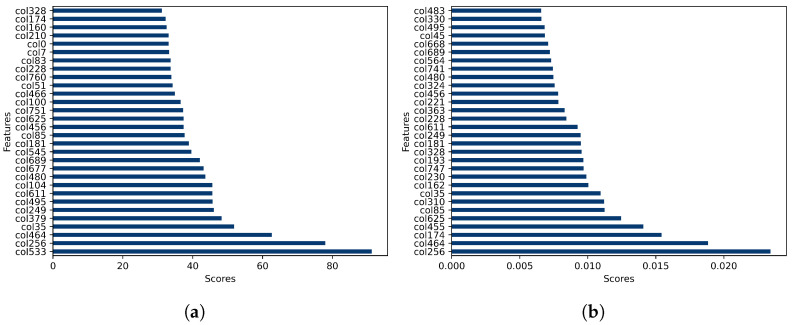
(**a**) Results of univariate feature selection and (**b**) feature importance analysis using extra trees classifier.

The feature selection process employed three distinct algorithms: univariate selection, extra trees classifier, and SHAP values. Univariate feature selection is a technique widely used in machine learning and data analysis, identifying the most relevant feature indices from a dataset based on their individual statistical properties, as illustrated in Figure 3a. Additionally, the extra trees classifier is utilised to extract important features from the dataset, providing insights into the relative importance of each feature and their impact on model predictions. Figure 3b displays the feature importance scores obtained from the extra trees classifier, highlighting each feature’s contribution to predicting the target variable.

**Figure 4:**
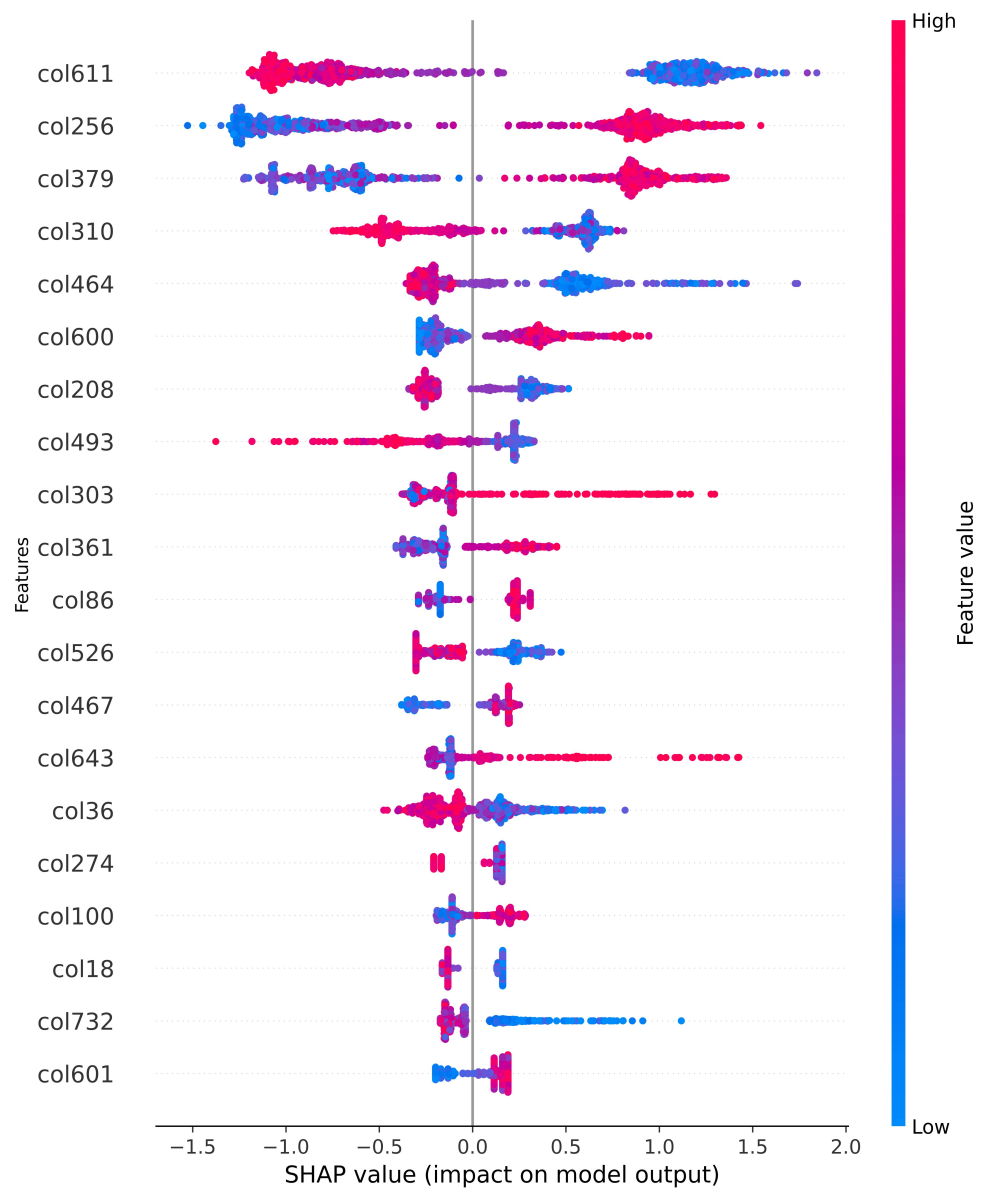
Shapley additive explanations (SHAP): the SHAP values most impactful on features of the model.

Furthermore, Shapley additive explanations (SHAP) values are leveraged to emphasise the feature selection process. SHAP values offer a comprehensive understanding of feature importance in machine learning models, providing insights into the contribution of each feature to individual predictions. Unlike traditional methods, SHAP values offer a nuanced perspective on feature importance, enabling practitioners to identify influential features and their impact on specific predictions. This level of granularity enhances interpretability and trust in model predictions, ultimately improving decision-making processes [20,21]. Figure 4 visualises the influence of each feature on predictions, playing a crucial role in interpreting the results.

By utilising these approaches, the selection of the most relevant features is ensured. Out of the 767 columns generated as text embeddings, the first 30 most-relevant columns were extracted for further processing, as shown in Table 1.

### 3.3. Bidirectional Encoder Representations from Transformers

BERT is a transformer-based model developed by Google AI that is designed to generate deep bidirectional representations of input text. The model consists of an encoder architecture that processes input sequences in both forward and backward directions, allowing it to capture contextual information effectively [12]. BERT produces two primary outputs: embeddings and attention scores. In embeddings, BERT generates contextual word embeddings for each token in the input sequence. These embeddings capture the semantic meaning of each token in the context of the entire input sequence. In addition, BERT also produces attention scores that indicate the importance or relevance of each token to every other token in the input sequence. These attention scores are represented as a matrix *A*, where $A_{ij}$ denotes the attention weight assigned to token *i* when processing token *j*.

### 3.4. Tokenisation and Embedding Extraction for Histopathology Reports

To analyse histopathology reports using BERT, the following steps are typically followed. Histopathology reports are first tokenised into individual words or subword units using BERT’s tokeniser. This process involves breaking down the text into tokens and mapping them to corresponding indices in BERT’s vocabulary. After tokenisation, the tokenised sequences are fed into the BERT model, which generates embeddings for each token in the input sequence, capturing the contextual information of the entire report. The embeddings from the last hidden state layer of BERT are often used for downstream tasks as they contain rich contextual information. Additionally, attention scores can be extracted to understand the relationships between tokens in the report. Once embeddings are obtained for all tokens in the report, the mean embeddings are calculated for all tokens. Mathematically, this can be represented as

(1) $$\mathrm{Mean}\;\mathrm{Embedding}=\frac{1}{N}\sum_{i=1}^{N}E_{i},$$

where *N* is the total number of tokens in the report and $E_{i}$ represents the embedding for token *i* [12,22].

### 3.5. Building Attentions per Node in the Dataset

Considering the varying lengths of histopathology reports, padding and truncation parameters are applied to represent them as fixed-size feature vectors. Padding ensures that shorter sequences match the length of the longest sequence by adding special tokens, while truncation cuts longer sequences to a predefined length. This standardises the length of all sequences [23]. Each report is processed to extract 767 features, utilising the embeddings from the last hidden state layer of BERT, which encapsulate rich contextual information [12,22]. These embeddings for each token are concatenated or aggregated to form a feature vector of length 767. Mathematically, the feature vector $F_{i}$ for token *i* can be represented as

(2) $$E_{i}=\left[E_{i1},E_{i2},\ldots ,E_{iN}\right],$$

where $E_{ij}$ represents the (*j*-th) dimension of the embedding for token *i*, and *N* is the total number of dimensions in the embedding space (e.g., 768 for BERT-base).

### 3.6. Refining Attention Mechanisms

BERT typically utilises multiple attention heads to capture different aspects of contextual relationships between tokens. The attention scores from each attention head can be aggregated by taking the mean across all heads to obtain a single attention score for each token. This process is repeated for each token in the report, resulting in a matrix of attention scores with dimensions (number of tokens) × 767. Mathematically, the attention score $A_{ij}$ for token *i* and dimension *j* can be calculated as

(3) $$A_{ij}=\frac{1}{H}\sum_{h=1}^{H}A_{ij}^{\left(h\right)},$$

where *H* is the total number of attention heads, and $A_{ij}^{\left(h\right)}$ is the attention score for token *i* and the dimension *j* from attention head *h*.

#### 3.6.1. Graph Construction

The initial phase of graph construction entails calculating the edges connecting nodes based on predefined criteria. This foundational step holds utmost significance, laying the concept for establishing meaningful relationships among medical records within the network by using the combined attributes and their attentions (the attention mechanism in the BERT output refers to the ability of the model to selectively focus on relevant parts of the input text when making predictions. It assigns different weights to each word or token in the input sequence, allowing the model to prioritise important information and ignore irrelevant parts. This attention mechanism helps BERT achieve better understanding and performance in natural language processing tasks) [24] contained within each node to explore the complexity of the connections that define the adjacencies between nodes in the graph. By computing attention across all inputs within each observation using Algorithm 1, the ascertained probabilities are assigned to each node’s classification as either 0 (no metastasis) or 1 (metastasis patient).
**Algorithm 1** Calculate the output for report**Input:** text ▹ List of all Histopathology reports**Input:** df ▹ Data frame**Output:** attentions, embeddings  1: **for** each **text** ∈ **df**[‘Report’] **do**  2:      add text tokinisation to the output  3:      add text attention to attentions  4:      add text embedding to embeddings  5: **end for**  6: **return** attentions, embeddings

By computing attention scores for all features in each node using Algorithm 1, the probabilities associated have been obtained by classifying each node as 0 (no metastasis) or 1 (metastasis). Additionally, it examines the connections between neighbouring nodes based on their respective attention scores using Algorithm 2. For instance, it indicates that a node should be linked to a group where the average attention score is either less than or equal to a predefined threshold. The threshold is fine-tuned experimentally by systematically varying its value and evaluating the model’s performance across different thresholds. This involves selecting a range of threshold values and then training and testing the model with each value to observe how it affects the model’s performance metrics, such as accuracy, precision, recall, or F1 score. Finally, by determining all of these criteria, the adjacency metric building comes into place in Algorithm 3.
**Algorithm 2** Calculate the attentions per node**Input:** attentions ▹ attentions**Output:** attention scores per node  1: **for** each **attention matrix** ∈ **attentions do**  2:      Stacked all the layers per node  3:      Find the average attention per node  4: **end for**  5: **return** attention scores per node

**Algorithm 3** Get node adjacents
**Input:** df ▹ Data frame
**Input:** attention ▹ Node attention
**Input:** cls ▹ Node class
**Input:** node threshold = 200
**Input:** node connections = 5
**Output:** adjacentsArray
  1: **for** each **row** ∈ **df do**
  2:     **if** cls = 0 **and** result = 0 **then**
  3:         add attention to the zeroes dictionary
  4:     **end if**
  5:     **if** cls = 1 **and** result = 1 **then**
  6:         add attention to the ones dictionary
  7:     **end if**
  8: **end for**
  9: **if** cls = 0 **then**
  10:     Sorting zeroes dictionary
  11:     dic ⟸ zeroes dictionary
  12: **else**
  13:     Sorting ones dictionary
  14:     dic ⟸ ones dictionary
  15: **end if**
  16: **for** each i,val in dic **do**
  17:     **if** (attention - val) ≤ node threshold **then**
  18:         **if** adjacents ≥ node connections **then**
  19:            Exit
  20:         **end if**
  21:         add the node to the adjacency matrix
  22:         increment node counter
  23:         adjust the total mean of nodes
  24:     **end if**
  25: **end for**
  26: **return** adjacentsArray


**Figure 5:**
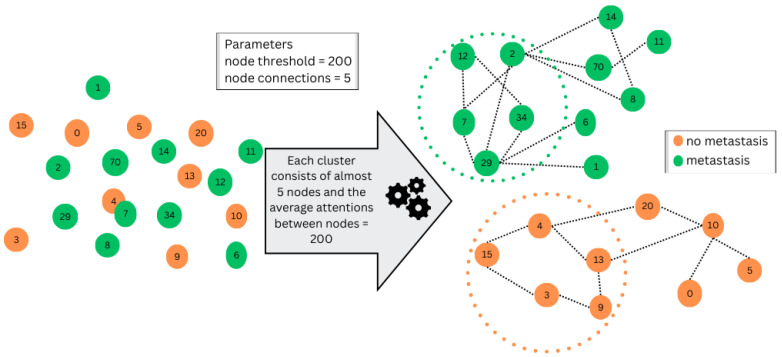
This diagram depicts connecting a node by considering the information from its neighbouring nodes. The figure highlights that the cluster information is influenced by both the node threshold value and the requirement that the number of nodes in each cluster not exceed the node connections parameter.

Based on these evaluations, the threshold value that yields the best overall performance or achieves the desired balance between different evaluation metrics is chosen as the optimal threshold. This threshold serves to establish connections between entities based on their similarities, facilitating the grouping of entities according to their attention levels. This threshold, which is an integer parameter, is integrated into the node grouping process. To determine whether a new node should connect to a group, a comparison should be made to find out the difference between the group’s average attention scores and the new node’s attention score against the threshold. When the disparity is within or equal to the set threshold and the cumulative count of nodes within the group does not exceed the permitted group size, the new node is linked to the existing group, as illustrated in Figure 5.

#### 3.6.2. Feature Extraction

The BERT model has revolutionised the analysis of textual data, including histopathology reports in the domain of medical diagnosis. The process involves tokenising the raw text of histopathology reports, converting it into a format that can be understood by machine learning models. BERT, being a state-of-the-art language model, is particularly effective in capturing the contextual information and semantic meaning of the text [12].

After tokenising the histopathology reports with BERT, the model produces embeddings for each token, encapsulating their semantic context. These embeddings serve as input features for subsequent tasks, such as classification. In the context of histopathology reports, these embeddings convert the text data into a high-dimensional space, capturing the intricate relationships and patterns. These embeddings are particularly beneficial for predicting BCM. However, given the substantial number of embeddings generated (in this instance, 767 columns), spotting the most informative features to guarantee precise prediction is essential. This is where feature selection techniques come into play. Three feature selection algorithms are employed, namely univariate selection, extra trees classifier, and the Shapely values, which are the most impactful features on the model, with many other methods that could be used like recursive feature elimination [25]. These algorithms analyse the embeddings generated by BERT and select the most relevant features that contribute significantly to the predictive performance of the model. The aim is to reduce the dimensionality of the feature space while retaining the most discriminate information. The selected features are then used to build the BG-MBC model. In this model, each patient number acts as an entity in the graph and is considered a node in the graph, and the selected features serve as node attributes. The attentions obtained from BERT are utilised to build the adjacency matrix, capturing the relationships between different medical records based on their semantic similarities and contextual dependencies, as explained in the algorithms mentioned above. This approach effectively leverages the power of BERT embeddings and attention mechanisms to extract meaningful information from histopathology reports and build a predictive model for cancer metastasis. By incorporating GNNs, the model can capture complex relationships and dependencies present in the data, leading to improved predictive performance [12].

### 3.7. BG-MBC Model

**Figure 6:**
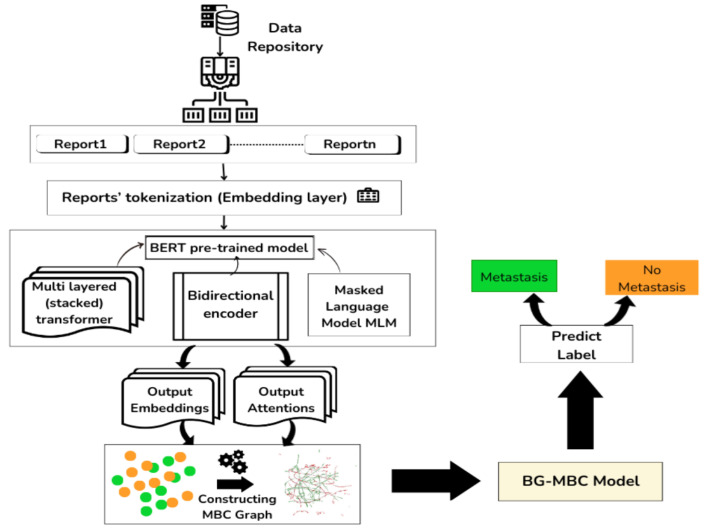
The diagram illustrating the process of utilising a BERT model to generate embeddings and attentions from histopathology reports. The embeddings and attentions are then used to construct a graph representation of the data. This graph is then passed to a BG-MBC model, which integrates GNN techniques for BCM prediction based on the structured data derived from the BERT model outputs.

The BG-MBC model architecture is a type of neural network that is specifically designed to operate on graph-structured data. It leverages GNN techniques to process and analyse the relationships and dependencies present in the data. The BG-MBC model architecture is summarised in Figure 6.

#### 3.7.1. Input Layer

The input to the BG-MBC model consists of a graph representation of the data, where each node in the graph corresponds to a data point (e.g., a medical record in the context of metastasis breast cancer diagnosis). The features associated with each node include information derived from embeddings and attentions obtained from pre-processing steps, such as those generated by a BERT model from histopathology reports.

#### 3.7.2. Graph Convolutional Layers

The core component of the BG-MBC model is the graph convolutional layers. These layers apply convolutional operations directly to the graph structure, allowing the model to aggregate information from neighbouring nodes. This enables the model to capture the local structure and dependencies within the graph, essential for tasks such as node classification or regression. The node classification layer of the BG-MBC model is crucial in the context of breast cancer prognosis. At the output of the model, this layer is responsible for assigning a label to each node in the graph, which suggests a positive or negative diagnosis of breast cancer metastasis. Typically, the node classification layer employs softmax activation to output probability distributions over the possible classes. During this stage, the model utilises two convolutional layers to aggregate information from node neighbours and nodes based on various options. In the aggregation phase, the model offers three options for updating node state. One option involves computing the sum of elements from a tensor along segments specified by a segment indices tensor, resulting in an aggregated message of type “sum”. Alternatively, the model can calculate the mean or maximum of elements for aggregation. The aggregated message is then used to update node information.

#### 3.7.3. Output Layer

The output layer serves as the concluding segment of our GNN-based node classification model, refining the learned representations and producing the ultimate node predictions. It consists of three different layers: batch normalisation, dropout, and dense with GELU activation. The output layer enhances the model’s predictive capabilities.

#### 3.7.4. Training Procedure

The BG-MBC model is trained using a supervised learning approach, where the model learns to predict the correct labels for the nodes in the graph based on the input features and the graph structure. The training process involves optimising a loss function, such as sparse categorical cross-entropy loss, using Adam optimisation.

#### 3.7.5. Interpretability

One of the key advantages of the BG-MBC model architecture is its interpretability. By leveraging Shapley values, BG-MBC enhances the transparency and explainability of its predictions, allowing the model to identify the importance of various features in predicting metastasis. This insight enables clinicians and researchers to understand the key factors influencing the predictions, thus providing a deeper understanding of the underlying drivers of metastatic breast cancer. Furthermore, analysing the learned parameters and the information propagation through the graph reveals the features and relationships that contribute most significantly to the model’s predictions. This level of interpretability is crucial for metastasis prediction as it ensures clinical acceptance and trust by clarifying the reasoning behind the model’s decisions.

### 3.8. BG-MBC and LLM (BERT) Models Integration

Here, the focus is on the integration of the large language model (LLM) BERT with BG-MBC to develop a comprehensive framework for analysing histopathology reports and predicting metastatic breast cancer, as described in Figure 6.

The last hidden layer in neural network models, especially in architectures like BERT, holds significant importance in various natural language processing tasks. In the context of analysing histopathology reports for predicting metastatic breast cancer, understanding the role of the last hidden layer becomes crucial for extracting meaningful insights [12,26]. In particular, the last hidden layer captures rich semantic information about the input text. It encodes contextualised representations of words and phrases, essential for understanding medical reports’ nuances and intricacies. This semantic representation forms the foundation for extracting relevant features and identifying patterns associated with metastatic breast cancer [27].

On the other hand, by analysing the activations and embeddings in the last hidden layer, it would be possible to extract important features that contribute to the classification of histopathology reports [28]. These features may encompass specific keywords, phrases, or contextual cues indicative of cancer metastasis. Extracting meaningful features from this layer enables the model to focus on the most relevant aspects of the input data [29]. The extraction of relevant features can be represented as

(4) F=ExtractFeatures(E),

where *E* denotes the embeddings generated by the BERT model for a histopathology report.

Moreover, BERT and similar models incorporate attention mechanisms, allowing them to dynamically weigh the importance of different words and context tokens in the input sequence. The attention scores generated in the last hidden layer highlight the salient aspects of the input text, guiding the model’s decision-making process. Understanding these attention patterns helps in identifying the key components of histopathology reports that contribute to the prediction of metastatic breast cancer [22]. The calculation of mean attention scores across layer heads is given by

(5) $$\bar{A}=\frac{1}{H}\sum_{i=1}^{H}A_{i},$$

where *A* represents the attention scores obtained from the BERT model, and *H* is the number of layer heads. On the other hand, in order to calculate the attention means per node that is considered, the below calculation can be applied:

(6) $${\mathrm{Attention}}_{\mathrm{node}}=\frac{1}{H}\sum_{i=1}^{N}{\mathrm{Attention}}_{{\mathrm{layer}}_{i}},$$

where ${\mathrm{Attention}}_{\mathrm{node}}$ represents the attention mean score for a particular node, *H* is the number of layers, and ${\mathrm{Attention}}_{{\mathrm{layer}}_{i}}$ denotes the attention score for the *i*-th layer.

Finally, in many cases, fine-tuning or transfer learning techniques are applied to pretrained models like BERT. The last hidden layer serves as a crucial component during these processes as it contains the most refined representations learned from extensive pretraining on large texts of histopathology reports. Fine-tuning allows the model to adapt its parameters to the specific task of predicting cancer metastasis, leveraging the knowledge encoded in the last hidden layer [30,31].

We analyse the histopathology reports using the BERT model to generate embeddings, from which relevant features are extracted to provide insights into the contents of the reports. This involves identifying key variables or dimensions within the embeddings that are indicative of metastatic breast cancer. Additionally, the extraction of important attention involves focusing on the last hidden state layer of the BERT model to extract important attention scores from each report. For each embedding variable, calculating the mean across all layer heads yields a comprehensive measure of attention for each report. This process enables us to quantify the relevance of different parts of the histopathology reports.

### 3.9. Graph Building

We use the embeddings and attention scores to construct a unidirectional and homogeneous graph. Each medical record serves as a node in the graph, with edges representing relationships based on attention scores between reports. This graph encapsulates the structural and semantic information present in the data. The construction of the graph involves defining nodes and edges based on attention scores.

(7) G=(V,E),

where *V* represents the set of nodes (medical records) and *E* represents the set of edges (connections based on attention scores).

### 3.10. Baseline Model

The baseline model comprises two feedforward networks implemented using Keras deep learning framework. Each network consists of a Keras sequential model composed of three stacked layers: batch normalisation, dropout, and dense layers with GELU activation function. These network layers are interconnected via skip connection layers. The baseline model takes input data, which are fed into the first feedforward layer, processed through the Keras sequential model, and then passed to the second feedforward network. The two networks are linked together by skip connection layers. This process is iterated four times, resulting in a Keras model with output logits.

### 3.11. BG-MBC Model Architecture

**Figure 7:**
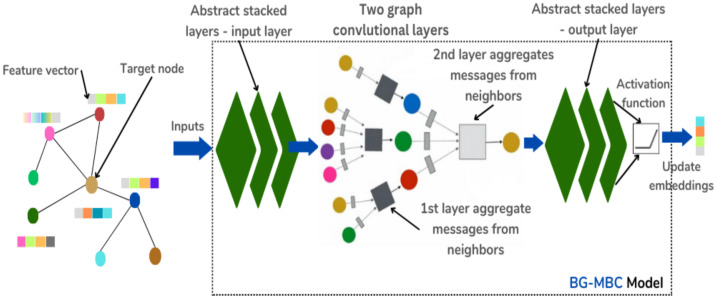
BG-MBC model architecture. The model consists of three main layers: the first and last layers are feedforward network layers, each containing three stacked layers. In between these feedforward layers, there are two graph convolutional layers responsible for generating and aggregating messages, which are then used to update the target node embeddings.

The output graph is passed into the BG-MBC model, which is a GNN architecture designed for node classification tasks. In Figure 7, the BG-MBC model begins with a pre-processing layer (input layer), which includes operations such as batch normalisation, dropout, and dense layers. These operations help standardise and prepare the graph data for further processing. Following the pre-processing layer, the BG-MBC model incorporates two graph convolutional layers. These layers aggregate and update node messages by considering information from neighbouring nodes and learning representations that capture the graph’s structure and semantics. Finally, the output from the graph convolutional layers passes through a post-processing layer (output layer), which includes batch normalisation, dropout, and dense layers. These operations refine the node representations and prepare them for the final node classification task.

(8) Y=BG-MBC(X).

where *X* represents the input data (graph) and *Y* represents the output (node classifications).

The BG-MBC model consists of pre-processing, graph convolutional, and post-processing. The operation of the graph convolutional layers involves aggregating and updating node messages as follows:

(9) $$H_{new}^{\left(l\right)}=\mathrm{GCNLayer}\left(H^{\left(l\right)}\right),$$

where $H^{\left(l\right)}$ represents the node representations at layer *l* and $H_{new}^{\left(l\right)}$ represents the updated node representations. The post-processing layer applies additional transformations to the node representations before the final classification, as follows:

(10) $$H_{final}=\mathrm{PostprocessingLayer}\left(H_{new}^{\left(L\right)}\right),$$

where $H_{final}$ represents the final node representations.

By integrating LLM BERT embeddings and attention scores with BG-MBC, a robust framework was created for analysing histopathology reports and predicting metastatic breast cancer. This approach leverages the strengths of both techniques and provides a holistic understanding of the underlying data, leading to improved diagnostic accuracy and patient outcomes.

## 4. Experimental Results

The experimental results showcase the effectiveness of the approach applied in predicting metastasis in breast cancer patients. This section encapsulates the data sources utilised and the diverse processes and tools employed to prepare the data for subsequent analysis.

### 4.1. Comparison with Existing Approaches

This work compares the proposed approach with three existing methods in the literature, in addition to the baseline model discussed in the previous section. The first method, which was proposed by Ting et al. [32], focused on convolutional neural network (CNN) enhancement for breast cancer classification. They utilised CNN architecture for classification tasks but did not explicitly incorporate graph-based representations or attention mechanisms. The second method, proposed by Thwin et al. [33], involved an attention-based ensemble network for breast cancer classification. They employed ensemble techniques along with attention mechanisms to improve classification performance. The third method, proposed by Mullooly et al. [34], applied CNNs to analyse breast biopsies and correlate tissue characteristics with mammographic breast density. While attention mechanisms were utilised in the second method, our method specifically focuses on leveraging BERT embeddings and attentions to construct a graph representation of histopathology reports. This approach enables a deeper understanding of the relationships between entities within the data.

### 4.2. Performance Evaluation

The dataset is partitioned into training (80%), and testing (20%). The Lambda Callback package is utilised to determine the optimal learning rate value. By monitoring loss values during training with various learning rates, the best learning rate value is identified in Table 2. The BG-MBC model parameters were determined through a systematic approach involving empirical testing, cross-validation, and best practices from the existing literature. Each parameter was selected to optimise the model’s learning efficiency while minimising overfitting and ensuring robust generalisation to new data.

To determine the optimal learning rate, the Lambda Callback package was utilised to record loss values during training. By analysing the resulting graph, the optimal learning rate was identified as the rate at which the loss decreased most rapidly before diverging or oscillating, which was between 1.2 × $10^{-2}$ and 1.3 × $10^{-2}$.

For the dropout rate, the literature commonly suggests starting with values between 0.2 and 0.5. Empirical testing within this range showed that a dropout rate of 0.2 provided the best balance between model complexity and performance.

Early stopping was employed to prevent overfitting by halting the training process when the model’s performance on a validation set ceased to improve. This ensures the model does not over-train on the training data and maintains its ability to generalise to new data.

Additionally, the model included two unique parameters: node threshold and node connections, which manage the relationships among nodes. These parameters were fine-tuned through trial and error to achieve optimal performance.

Finally, BG-MBC performance is assessed by using a set of evaluation criteria, including receiver operating characteristic (ROC) graphs between true positive rate and false positive rate, area under ROC curve (AUC), F1 scores, accuracy, and the cross-validation metric.

Overall, this comprehensive approach to parameter selection, including the use of cross-validation and empirical testing, ensured that the BG-MBC model achieved high accuracy and generalisability in predicting metastatic breast cancer.

### 4.3. Results

Table 3 shows the performance of the proposed approach and existing methods for metastatic breast cancer prediction using histopathology reports. It could be observed that the proposed approach, BG-MBC, achieves the best performance compared to existing methods. In particular, the BG-MBC achieves an accuracy score of 0.98. The reason why it outperformed existing methods is that it incorporates advanced graph-based techniques, such as GNNs and large language models, in constructing the graph. Moreover, it effectively captures the relationships and dependencies within the data. The model’s superior performance suggests that it can better model complex patterns and make more accurate predictions compared to existing methods. On the other hand, the baseline model achieves an accuracy score of 0.94. Although still achieving decent performance, it is short compared to BG-MBC, indicating that there is room for improvement in terms of capturing the underlying data structure and relationships. CNNI_BCC [32] achieves an accuracy score of 0.94, which is slightly lower than BG-MBC but still competitive. On the other hand, the model of Thwin et al. [33] achieves an accuracy score of 0.90, indicating that it may not capture the underlying patterns as effectively as BG-MBC or CNNI_BCC. Finally, the deep convolutional neural network model [34] achieves an accuracy score of 0.93, which is slightly lower than BG-MBC but still relatively good.

**Figure 8:**
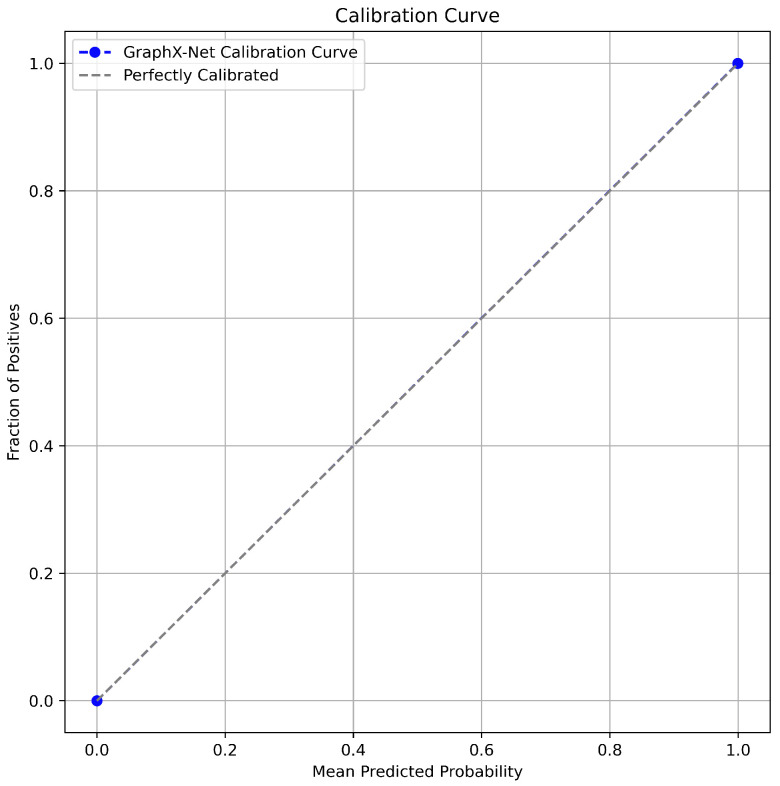
BG-MBC calibration curve demonstrating the alignment with the 45-degree diagonal line, indicating perfect calibration between predicted probabilities and actual outcomes.

Figure 8 shows the BG-MBC calibration curve. The calibration curve is a valuable tool used in machine learning to assess the reliability of the predicted probabilities of a model. It compares the predicted probabilities of a binary classifier with the actual observed probabilities. Calibration curves offer information on the consistency and accuracy of model predictions, helping to identify potential biases or overconfidence. They are particularly useful in applications where accurate probability estimates are essential, such as medical diagnoses or risk assessments [35]. As a result, Figure 8 suggests that the model’s probability estimates are trustworthy and can be used confidently for the metastatic breast cancer task and to make decisions based on probabilities. The alignment indicates that the model’s predicted probabilities are reliable and well-calibrated. When using these probabilities to make decisions or set thresholds, it is expected that the model’s predictions are accurate and representative of the true underlying probabilities.

## 5. Discussion

The combination of BG-MBC and large language models (LLMs) like BERT offers a promising approach for gaining insights from histopathology reports in the context of metastatic breast cancer diagnoses. By leveraging the strengths of both techniques, this integration enhances our understanding of the underlying data and improves predictive accuracy. The BERT model is capable of capturing the semantic meaning of each token in the context of the entire input sequence (histopathology reports) by utilising the attention scores obtained from the BERT model and extracting valuable information about the relationships between different tokens and phrases within the reports. The attention scores from the BERT model serve as a foundation for constructing the graph representation of the data. By considering the attention weights between tokens, it would be easy to identify the important connections and dependencies within the text. This graph representation allows us to capture the complex relationships between different features and their impact on the diagnosis of metastatic breast cancer. Incorporating node threshold attributes and connections in building the graph determines the significance of each node, while the connections between nodes reflect the relationships and dependencies between reports. By setting appropriate thresholds and establishing connections based on attention scores, the graph accurately represents the underlying structure of the data. The embeddings generated by the BERT model provide a rich representation of the semantic meaning of each token in the histopathology reports by using these embeddings as node features in the graph and incorporating the contextual information captured by the LLM into the GNN model. This enhances the model’s ability to understand the relationships between different features and make accurate predictions. As a limitation of this work, histopathology reports offer a static view of tissue characteristics, potentially missing dynamic changes crucial for accurate diagnosis. Moreover, interpretation can be subjective, leading to inconsistencies among pathologists and potential misclassification. Consequently, integrating histopathology data with other clinical information could address some of these limitations, enhancing the accuracy and reliability of the classifier.

## 6. Conclusions

Using histopathology reports to build a machine learning classifier for metastatic breast cancer (MBC) diagnosis offers several potential benefits. First, these reports provide detailed and standardised information about tissue samples, making them valuable for diagnosis. Second, leveraging histopathology data with advanced machine learning algorithms has the potential to achieve high accuracy in prediction tasks. In this paper, we introduce a hybrid mode using graph neural network (GNN) and bidirectional encoder representations from transformers (BERT) for MBC prediction, which we called BG-MBC, to analyse histopathology reports. By leveraging the complementary strengths of both techniques, this approach can improve our understanding of the disease and contribute to more effective diagnostic and treatment strategies in the medical field. In this study, we introduce a novel hybrid model, BG-MBC, which integrates graph neural networks (GNNs) and bidirectional encoder representations from transformers (BERT) to analyse histopathology reports for MBC prediction. The BG-MBC model leverages the unique strengths of both GNN and BERT to improve our understanding of metastatic breast cancer and foster more effective diagnostic and treatment strategies.

The following is a summary of the novelty and practical impact of the work:

1. Combining GNN and BERT: The integration of GNN and BERT is innovative in the context of medical data analysis. BERT excels at understanding the semantic relationships within text data, while GNN is adept at capturing the structural relationships in graph-structured data. By combining these two approaches, BG-MBC can utilise both the linguistic context from the histopathology reports and the intricate connections between different medical features.
2. Enhanced prediction accuracy: By leveraging BERT’s ability to capture the semantic meaning of the text and GNN’s capacity to model relationships and dependencies between data points, BG-MBC provides a more holistic analysis. This dual approach leads to higher prediction accuracy, making it a powerful tool for MBC diagnosis.
3. Comprehensive data representation: The model uses BERT to generate embeddings that capture the semantic richness of the text in the reports. These embeddings are then used as node features in a graph constructed based on the attention scores, reflecting important connections within the text. This comprehensive representation enhances the model’s understanding of the complex interplay between different medical features.
4. Improved understanding of disease: By accurately modelling the relationships and dependencies within the data, BG-MBC can provide deeper insights into the underlying patterns and factors contributing to metastatic breast cancer. This understanding can guide clinicians in making more informed decisions regarding diagnosis and treatment.

In summary, the BG-MBC model represents a significant advancement in the field of medical diagnostics by combining the complementary strengths of GNN and BERT. This innovative approach not only enhances predictive accuracy but also provides valuable insights into the disease, ultimately contributing to more effective diagnostic and treatment strategies. Future research can further explore the potential of this approach by investigating different LLM architectures, refining the graph construction process, and evaluating the model’s performance on larger and more diverse datasets.

## Figures and Tables

**Table 1:** Experiment findings on feature selection.

Illustration	MBC Cross-Sectional Data
Size of the training set	813
Size of the test set	187
Total class count	2
Total number of features in the dataset	767
Number of training data features after correlation	30
Number of nodes	1000
Number of node features in the optimal set	30
Number of edges	4984

**Table 2:** Values of BG-MBC model hyperparameters.

Model Hyperparameters	Value
hidden units	[34, 34]
learning rate	0.01
dropout rate	0.2
num epochs	300
batch size	128
k-fold	3

**Table 3:** Detailed evaluation metrics breakdown of BG-MBC and existing methods. Best results are highlighted in bold, and second-best results are underlined. All scores given as ‘-’ are not provided in their sources.

Models	AUC Score	F1 Score	Balanced Accuracy	Cross-Validation
BG-MBC	**0.98**	**0.98**	**0.98**	**0.99**
Baseline model	0.90	0.90	0.94	0.92
CNNI_BCC [32]	0.90	-	0.90	-
DL-CNN [33]	0.91	-	0.90	0.89
DCNN [34]	0.96	-	0.93	-

## Data Availability

All the data used in the research can be found in the reference [19].

## Abbreviations

The following abbreviations are used in this manuscript:

LLM	Large language model
GNN	Graph neural network
XAI	Explainable artificial intelligence
MBC	Metastatic breast cancer
BERT	Bidirectional encoder representations from transformers
